# Sensitivity analysis of SARS-CoV-2 aerosol exposure

**DOI:** 10.3205/dgkh000399

**Published:** 2021-10-07

**Authors:** Christian Redder, Christian Fieberg

**Affiliations:** 1 Delbag GmbH, Herne, Germany; 2 Westphalian University of Applied Sciences, Gelsenkirchen, Germany

**Keywords:** aerosol, airborne iInfection, SARS-CoV-2, ventilation, face masks, air hygiene

## Abstract

As vaccination campaigns are in progress in most countries, hopes to win back more normality are rising. However, the exact path from a pandemic to an endemic virus remains uncertain. While in the pre-vaccination phase many critical indoor situations were avoided by strict control measures, for the transition phase a certain mitigation of the effect of indoor situations seems advisable.

To better understand the mechanisms of indoor airborne transmissions, we present a new time-discrete model to calculate the level of exposure towards infectious SARS-CoV-2 aerosol and carry out a sensitivity analysis for the level of SARS-CoV-2 aerosol exposure in indoor settings. Time limitations and the use of any kind of masks were found to be strong mitigation measures, while how far the effort for a strict use of professional face pieces instead of simple masks can be justified by the additional reduction of the exposure dose remains unclear. Very good ventilation of indoor spaces is mandatory. The definition of sufficient ventilation in regard to airborne SARS-CoV-2 transmission follows other rules than the standards in ventilation design. This means that especially smaller rooms most likely require a significantly greater fresh air supply than usual. Further research on 50% group models in schools is suggested. The benefits of a model in which the students come to school every day, but for a limited time, should be investigated. In terms of window ventilation, it has been found that many short opening periods are not only thermally beneficial, they also reduce the exposure dose. The fresh air supply is driven by the temperature gradient and wind speed. However, the sensitivity towards these parameters is not very high and in times of low wind and temperature gradients, there are no arguments against keep windows open in order to make up for the reduced air flow rate. Long total opening periods and large window surfaces will strongly reduce the exposure. Additionally, the results underline the expectable fact that exposure doses will increase when hygiene and control measures are reduced. It seems advisable to investigate what this means for the infection rate and the fatality of infections in populations with partial immunity. Very basic considerations suggest that the value of aerosol reduction measures may be reduced with very infectious variants such as delta.

## Zusammenfassung

In vielen Ländern schreiten die Impfkampagnen voran und die Hoffnungen auf eine Rückkehr zu mehr Normalität steigen. Trotzdem bleibt die Entwicklung von SARS-CoV-2 von einem pandemischen hin zu einem endemischen Virus weiter ungewiss. Während vor Beginn der Impfkampagnen viele kritische Situationen durch strickte Maßnahmen unterbunden wurden, scheinen in der Übergangsphase zumindest in Innenräumen abhelfende Maßnahmen angezeigt.

Um die Mechanismen der Aerosolübertragung in Innenräumen besser zu verstehen, wird ein zeitdiskretes Modell zur Berechnung der Aerosol Exposition vorgestellt. Mit Hilfe des Modells wurde eine Sensitivitätsanalyse der Exposition in Innenräumen durchgeführt. Gesichtsmasken und eine zeitliche Limitierung von Ereignissen wurden als starke Kontrollmaßnahmen identifiziert. Ob der zusätzliche Aufwand eines obligatorischen Einsatzes von professionellen Masken in Alltagssituationen den Mehraufwand rechtfertigt, konnte nicht geklärt werden. Eine ausreichende Belüftung von Innenräumen ist zwingend notwendig. Die Definition einer ausreichenden Belüftung folgt in Hinblick auf SARS-CoV-2 Aerosol Infektionen jedoch anderen Regeln als sie sonst bei der Auslegung von Lüftungsanlagen angewendet werden. Das führt dazu, dass insbesondere kleine Räume unter Umständen eine signifikant höhere Frischluftzufuhr als sonst benötigen. Weitere Untersuchungen des Wechselunterrichts an Schulen werden vorgeschlagen. Die Vorteile eines Wechselmodells, bei dem beide Gruppen täglich aber für limitierte Zeit zum Unterricht kommen, sollten untersucht werden. Im Falle einer Fensterlüftung sind viele kurze Intervalle wenigen langen nicht nur aus thermischen Gründen vorzuziehen, sie reduzieren auch die Aerosolexposition. Die Frischluftzufuhr über Fenster wird von Wind und Temperaturunterschied angetrieben. Trotzdem ist die Sensitivität gegenüber diesen Parametern nicht extrem ausgeprägt. In Zeiten von schwachem Wind und geringen Temperaturunterschieden spricht nichts gegen eine dauerhafte Öffnung der Fenster. Lange Öffnungsperioden sowie große Fensterflächen führen jeweils zu einer starken Reduktion der Aerosolbelastung. Die Ergebnisse lassen erwartungsgemäß den Schluss zu, dass die Expositionsdosen zunehmen werden, wenn Kontroll- und Hygienemaßnahmen in der Breite reduziert werden. Welche Folgen das in einer Bevölkerung mit partieller Immunität hat, sollte weiter untersucht werden. Auf Basis rudimentärer Betrachtungen kann man, zumindest für moderate Aerosolreduktionsmaßnahmen, eine leichte Tendenz erkennen, dass die relative Wirkung solcher Maßnahmen bei der Verhinderung von Infektionen, für deutlich ansteckendere Varianten (wie z.B. Delta) etwas reduziert ist.

## 1. Introduction

Airborne transmission has been found to play a major role in the spread of SARS-CoV-2. Although health authorities such as the CDC claim that it is not subject to “true airborne infection”, the fact that accumulation of small infectious aerosol particles causes numerous infections in poorly ventilated indoor environments is no longer in doubt [1], [2], [3], [4], [5], [6]. Therefore, health authorities point out the need for proper ventilation [7], while engineers suggest technical solutions, e.g., air cleaning and modified HVAC operation for indoor environments [8], [9], [10]. The modelling of SARS-CoV-2 aerosol transmission has been subject to several studies. Müller et al. [11] use a linearized Wells-Riley model. A similar approach which suggests a linearized aerosol distribution model, with additional parameters, attempts to estimate the risk by calculating the exhaled, inhaled and deposited SARS-CoV-2 D50 dose [12]. Kriegel et al. [13] also suggest a Wells-Riley model to estimate the infection risk, using a more sophisticated aerosol model. Buonano et al. [14] uses a similar approach, applying a Monte-Carlo method for the risk assessment. However, the estimated dose required for an infection varies and still poses a considerable uncertainty. The sensitivity of the cumulated exposure dose to basic parameters of indoor environments can be calculated using relative doses only. Therefore, in order to provide practical advice for creating low-exposure environments in indoor settings, a sensitivity analysis was carried out here for varying parameters of indoor settings. Besides gaining a more intuitive understanding of the non-linear interrelations, the aim was to identify critical situations, impact factors and levels, and the capability and usefulness of mitigation measures.

## 2. Methods

The sensitivity of exposure towards SARS-CoV-2 aerosols in the presence of one infected person was investigated by modelling indoor situations. The model is described and all functional parameters were evaluated in order to meet the current state of research. In the first part of the simulations, a sensitivity analysis was carried out for general parameters of indoor environments, and in the second part specifically for window ventilation. The sensitivity analysis shows the relative change of the exposure compared to the change of fundamental environmental parameters, starting from a defined situation (zero situation). In order to assess the extent to which the results are affected by the specificity of the zero situation, the zero situation was shifted to the upper and lower limits of the first analysis. This was done for three parameter pairs.

### Description of the Model

**Figure 1:**
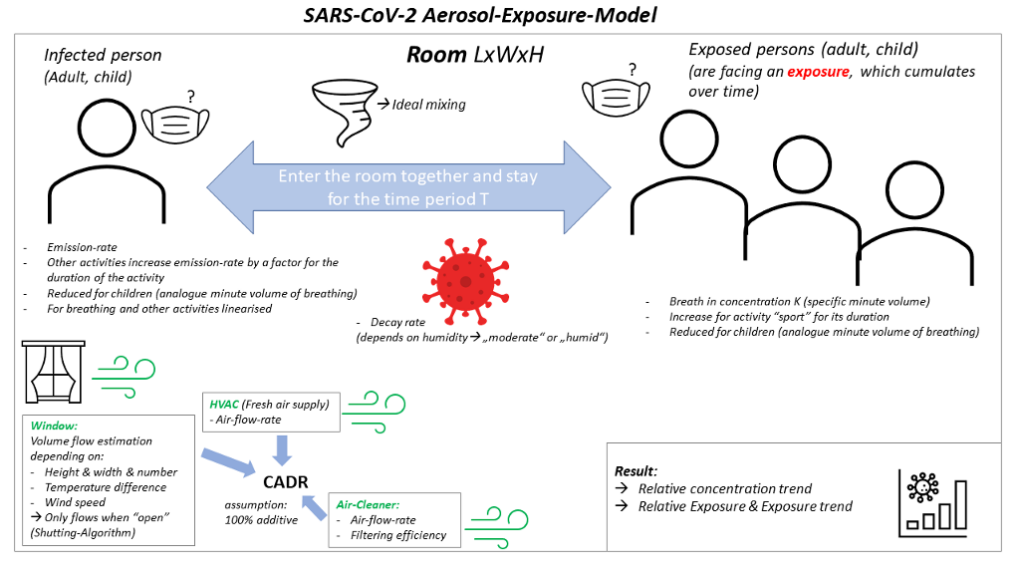
Illustration of the model

A discrete, transient unsteady time-step model was developed to describe the exposure dose in presence of one infected person. The model needs to be fed with input parameters defining the modelled situation, while pre-set functional parameters define the biological and technical setting. The parameters are processed in both static and dynamic equations, which are updated at every time or iteration step. The time steps are iteratively scaled down by a divisor until the difference of the exposure between the last two iterations falls short of the defined maximum value for the convergence. The general behavior of aerosol transport from person to person is close to the analytical function of a Wells-Riley model, but it allows the use of transient and discontinuous functions for the input parameters. The model can handle absolute doses (e.g., quantum dose), but for our calculations of sensitivity, relative doses were sufficient. Figure 1(Fig. 1) illustrates the function of the model.

**Figure 2:**
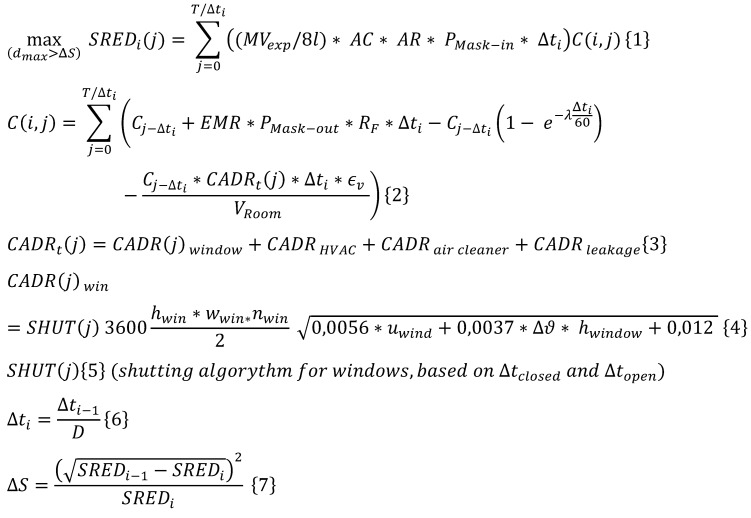
Dynamic model equations

**Figure 3:**
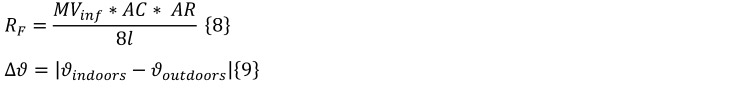
Static model equations

- Dynamic model equations
  For dynamic model equations, see Figure 2 (Fig. 2)

- Static model equations
  For dynamic model equations, see Figure 3 (Fig. 3)

- Output values
  For output values, see Table 1 (Tab. 1)

- Input parameter
  For input parameters, see Table 2 (Tab. 2)

- Functional parameter (pre-set)
  For functional parameters, see Table 3 (Tab. 3)

### Discussion of functional parameters 

***AC* – activity factor**

The activity factor for breathing, talking and singing refers to measured particle mean flows for certain activities as published in [15]. The AC for sports is calculated by the increased minute volume during sports (please refer to “minute volume”).

***CADR*** ***_leakage_*** **– leakage air flow of the building**

Every building has a natural air flow through leakages, providing additional fresh air. The values for this air flow are subject to high variations. While modern energy-optimized buildings have very low natural leakages, older buildings are not as air tight. For instance, Howard-Reed et al. [16] suggest a leakage of 0.37 air exchanges per hour. The value was measured from a 1986 wood and stucco house, which suggests that it is relatively high. Modern houses have significantly lower natural leakage rates, as they are often intentionally built to be air tight. However, in order to not neglect the leakage, a mid-range value of 0.15 air exchanges per hour was chosen.

***CADR_win_*** **– fresh air supply through windows **

The fresh air supply through windows is estimated by a model which was experimentally verified in [17]. The temperature-driven terms of the model showed a very good correlation to the measurements. The wind-driven term was not as accurate, because it only accounts for a scalar value for the wind velocity. However, for an interdisciplinary model like ours, this simplification seems advantageous and the inaccuracy acceptable. The indoor temperature is considered to be constant, which may cause some inaccuracy for very long opening times with cold outdoor temperatures. But this is not a desired or practicable way to ventilate rooms. The indoor temperature should be kept within a limited range in order to reduce the thermal stress of the users.

**
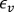
** **– ventilation efficiency**

Dilution ventilation, which typically has a ventilation efficiency of 1, is assumed for all three types of fresh air supply [18]. There are HVAC settings which have an even higher efficiency than 1, but these are probably not the standard in most of the considered everyday situations. In real-life situations, there is always a certain degree of air movement. Therefore, the value is unlikely to fall much below 1. However, poor positioning of air cleaners (e.g., under lamps) can reduce the efficiency [19], just like window-ventilated rooms with a depth approximately >3 times their height [20].

***MV*** **– minute volume**

The amount of inhaled particles correlates to the air volume breathed in by an exposed person. Therefore, people with smaller lungs (e.g., children) inhale fewer aerosol particles, while activities like sports increase the exposure. On the other hand, smaller lungs emit less aerosol, while sports increase the amount due to the increased minute volume. The data for different ages are based on [21], the value for sports on [22].

***P_Mask_***

The penetration of the masks needs to be considered in both directions: It reduces the aerosol emission of the infected person and the exposure of the healthy persons. The theoretical efficiency of professional masks (e.g., N95) is very high (technical potential >95%). However, in everyday use, the aging of the masks, tight wearing and discipline of the users reduce the efficacy. Besides the technical approach, epidemiological studies provide data for the real-life efficicacy of masks. Mitze et al. [23] suggest 40% efficacy when masks are obligatory in many everyday situations. Since such global approaches also include situations in which no masks are obligatory, the real-life efficacy for specific situations will probably be underestimated. For the model, an efficacy of 30% for both directions is assumed (P=0.7) as the pre-set condition. This means an overall efficacy of 51% (1–0.7*0.7). A good overview on the different aspects of masks is given in [24]. Obviously, the model considers a public health view rather than a personal view. The intelligent use of high efficiency masks can add much quality to personal protection and ensure effectiveness close to the technical potential.

*λ* **– decay rate of viable SARS-CoV-2 in aerosols**

For the viral activity of SARS-CoV-2 in aerosols, the principle described below is considered. An infected person exhales droplets (mucus, surfactant, saliva), which contain viable SARS-CoV-2 virions. The water content vaporizes at a rate depending on the droplets’ other contents and the surrounding relative humidity. As soon as the higher proportion of water content vs the residual matter has dropped to a certain level, the process may slow down. Under constant environmental conditions, each particle will at some point enter a state of equilibrium, at which point no more water vaporizes. Its main content is the residual matter and it is called a droplet nucleus. At higher humidity, droplets shrink at a substantially slower rate and can even enlarge by gaseous H_2_O from the air [25]. The survival of most pathogens strongly declines with the droplet size [26]; and the life-time within a droplet nucleus is limited. A laboratory study on MERS-Coronavirus allows a good reconstruction of the aforementioned cycle [27]. While dry conditions showed an intense shrinking of synthetic droplets with MERS-virus and a strong decay of viable virus, both were significantly slower under more humid conditions. A similar study was conducted on SARS-CoV-2, using synthetic droplets and artificial saliva for humid and moderate environmental conditions [28]. The decay rates found for artificial saliva in this study were used for our model. Two further studies, with non-variable environmental conditions, are in a range of accordance, which supports the correctness of these values [29], [30]. However, another source does not agree with these assumptions [31]. This study found significantly higher decay rates at high (70% RH) than at low humidity (20% RH). For low humidity, the decay was very slow at 20°C and at 30°C, no decay was found. 

**Figure 4:**
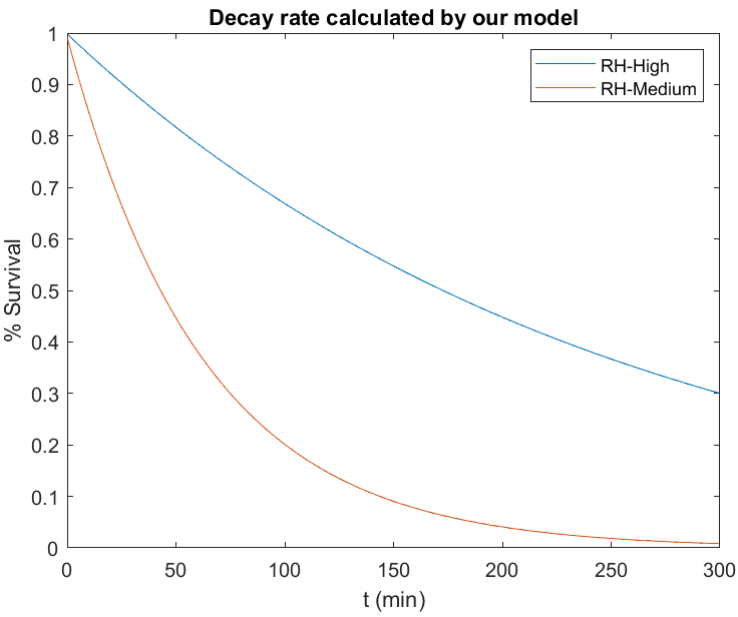
Decay of viable virus over time

In order to verify the decay function in the model, we applied it to a room with an initial SARS-CoV-2 concentration of 1, without a source function (no infected person at t=0). Figure 4 (Fig. 4) shows the decay over time, which is in very good accordance to the curves modelled in [28], according to their measurements. 

## 3. Results of the sensitivity analysis

### General parameters

**Figure 5:**
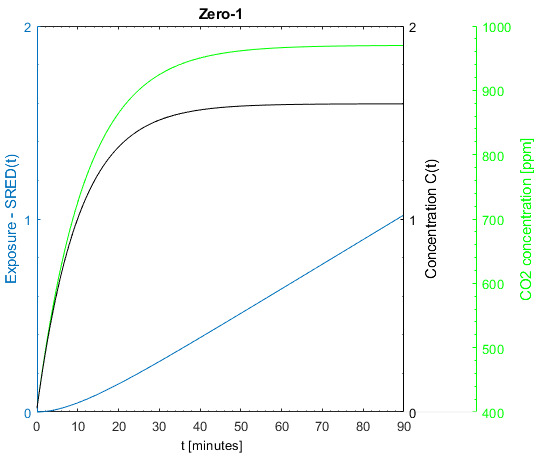
Change of exposure, virus- and CO_2_ concentration over time

For the general analysis, the relevant input values for the zero situation (starting point) are shown in Table 4 (Tab. 4). In terms of the room volume and no. of persons present, it comes close to classroom situations. The simulation results of the zero situation are shown in Figure 5 (Fig. 5). With the chosen CADR of 1,000m³/h, the CO_2_ concentration is kept well under 1,000 ppm. The ventilation in many real classes may be much worse. The concentration trend is typical for a constant fresh air supply. After a buildup phase, which lasts about 30 minutes in this case, the concentration of SARS-CoV-2 reaches equilibrium. While the increase of the cumulated exposure SHRED(t) increases exponentially in the steep build-up phase, the increase becomes linear during the equilibrium state. 

**Figure 6:**
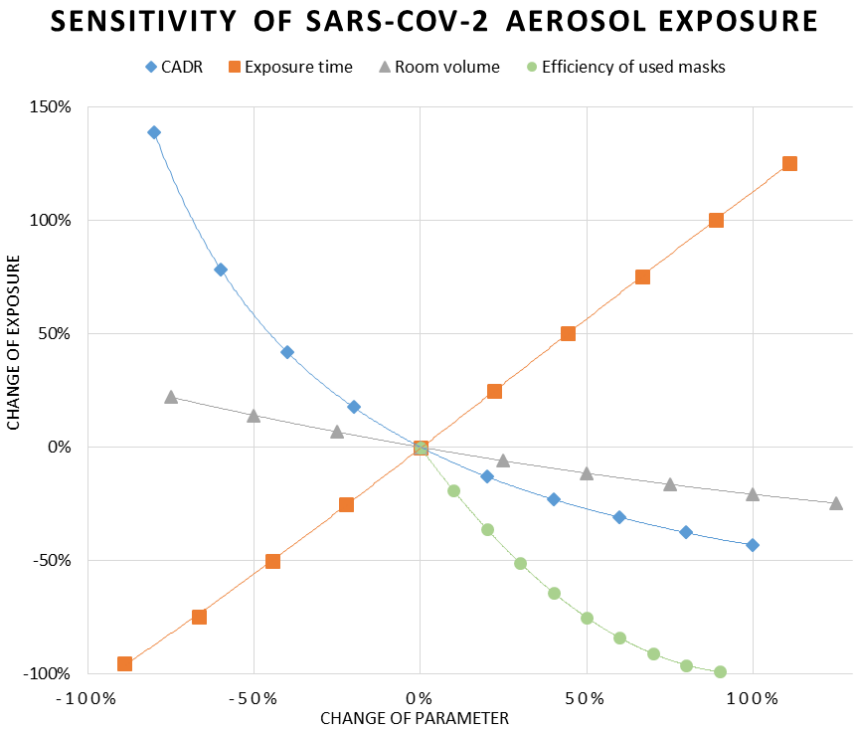
Sensitivity analysis for basic parameters

The results of the sensitivity analysis are shown in Figure 6 (Fig. 6), and the absolute numbers of the parameters tested for their sensitivity are shown in Table 5 (Tab. 5). They are on the same order as the data points from left to right on the x-axis of the figures. As already mentioned, the time dependence of SRED follows a linear function after the equilibrium concentration is reached. As long as any kind of constant fresh air supply is provided, an equilibrium state will be reached at some point. The gradient of linearity is always >1, as will be explained later. The exposure shows a log-decay in relation to the CADR. As the curve flattens for CADR higher than in the zero situation, the chosen air flow rate seems in an effective range for this setting. Larger rooms will reduce the exposure. The sensitivity for the room volume seems almost linear, but has a slight logarithmic curvature. The efficacy of masks increased for the infected and uninfected person in parallel, considering the same values for active and passive protection, which is a simplification. The fact that inhaling has the tendency to pull the mask into a tight fit, while exhaling does the opposite, may have some effect. On the other hand, the fresh droplets in one exhaled breath, which are oversaturated with humidity, will most likely be much larger on average. Although experts assume that the active and passive protection level of masks is in a similar range [32], comparable data for both directions are rare. 

An increase of mask efficacy causes a log-decay of the exposure. This means that wearing a mask at all, even with low efficacy, is very effective in reducing the exposure dose, while the curve flattens for higher efficacy. In spite of the fact that the theoretical technical potential of professional masks is >90%, the average real-life performance is likely to be significantly lower, as already discussed in the parameter evaluation. Therefore, the reduction values for 90% efficacy may only be possible in very professional settings, if at all. Besides the problem that highly effective masks absolutely require continuous checking of their tightness of fit in order to achieve their whole filtration potential, factors such as beards and differing facial forms facilitate leakages. Additionally, it must be considered that most professional masks are made of synthetic materials, which depend on their electric charge and their microstructure in order to achieve their specified efficacy. The re-use and long-term use of the same mask are likely to significantly reduce its efficacy. Therefore, rules demanding the strict use of professional masks should be critically questioned, since other factors such as costs and additional waste also need to be taken into account.

To gain better understanding on how much the sensitivity behavior is related to the specificity of the zero-situation, we tested how the sensitivities affect each other by shifting the zero situations to the maximum and minimum values of the parameter change for parameter pairs. The max. and min. values thus become the new zero points and the parameter and exposure change is shown relative to them (not the original 0.0). This means that for the lines not intersecting 0.0, the parameter changes need to be calculated by subtracting the (x, y) values from the connecting knot to the lines passing 0.0. 

Figure 6 (Fig. 6) shows the sensitivities for room volume and CADR. As can be seen in the diagram, the room volume has a linear impact at higher ventilation rates. At the lowest ventilation rate (200 m³/h), the exposure shows a log decay with room size. It also shows a log decay with increasing CADR, which is most distinct at small room volumes. This diagram points out how problematic small, poorly ventilated rooms are. As in buildings with HVAC systems, the CADR is normally calculated based on the expected number of people. Smaller rooms will naturally have smaller CADR rates and the lower, modelled CADR rates are thus realistic for smaller room volumes. Large rooms with ventilation systems designed for many people will thus normally promote low aerosol exposure. However, as mentioned above, log decay with increasing CADR for larger rooms still exists. This shows that large rooms can easily lose much of their advantage when poorly ventilated.

**Figure 7:**
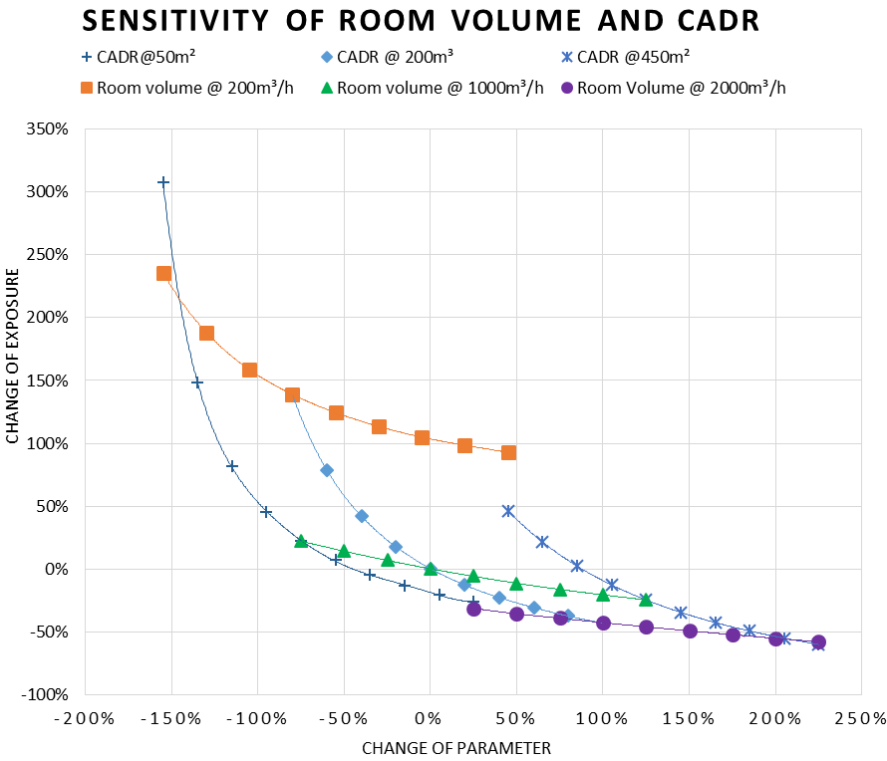
Sensitivity of room volume and CADR

**Figure 8:**
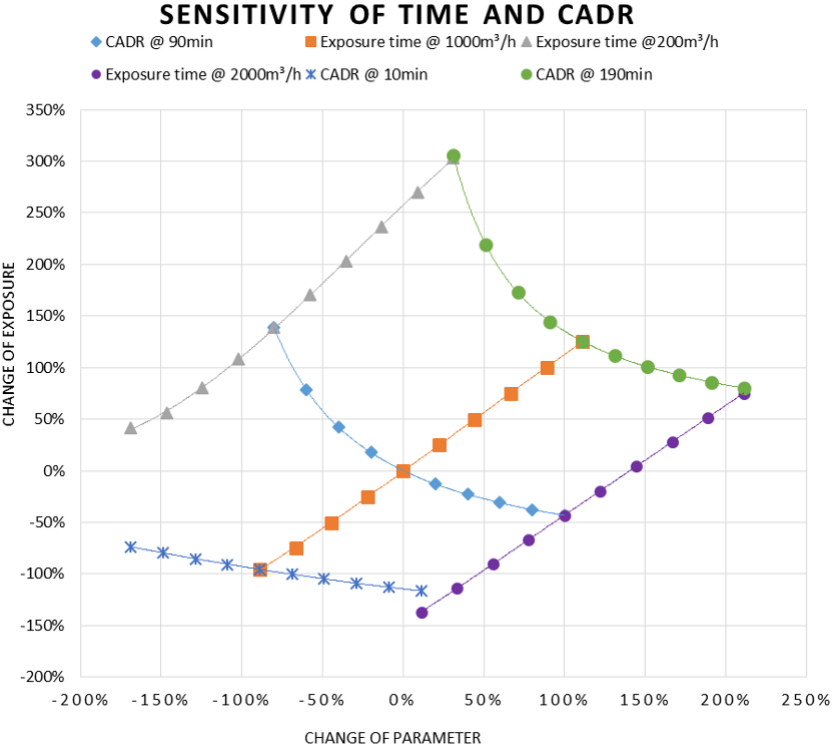
Sensitivity of exposure time and CADR

Figure 7 (Fig. 7) shows the sensitivity for CADR and exposure time. The time-related increase of the exposure can be divided into two parts. During the concentration build-up time, exposure increases exponentially until the equilibrium concentration is reached. The increase then becomes linear. This is most pronounced at 200 m³/h. For lower CADR, the build-up time is longer, leading to a longer exponential increase and a larger gradient of the linear increase. Since there is always a build-up time in which the exposure increases exponentially with a flat start, the gradient of the following linear phase of the time-based exposure increase is always >1. For the medium (1,000 m³/h) and higher CADR (2,000 m³/h), the exponential start is not very pronounced, because the build-up time is shorter and everything happens on a lower level. A detailed concentration build-up can be seen in Figure 4 (Fig. 4). Looking at the linear part of the lowest CADR (200 m³/h) curve, the gradient is >1.4. For poorly ventilated environments, this means that doubling the time leads to significantly more than twice the exposure. As the reason for this can be found in the long build-up time due to the low ventilation rate, all this happens with a relatively high level of exposure. This points out how important it is to consider the duration of events when planning infection control measures for SARS-CoV-2. Looking at Figure 8 (Fig. 8), a similar behavior can be found in large rooms. Due to their size, they have longer build-up times until the concentration reaches equilibrium. However, since the size also reduces the concentration level, all this happens at a lower exposure level.

The most conspicuous curve in Figure 8 (Fig. 8) is the one showing the change of exposure with changing room volume at 10 minutes exposure time. It shows a significant log decay with increasing room volume. However, due to the short time period, this happens at a relatively low level.

### Window ventilation

**Figure 9:**
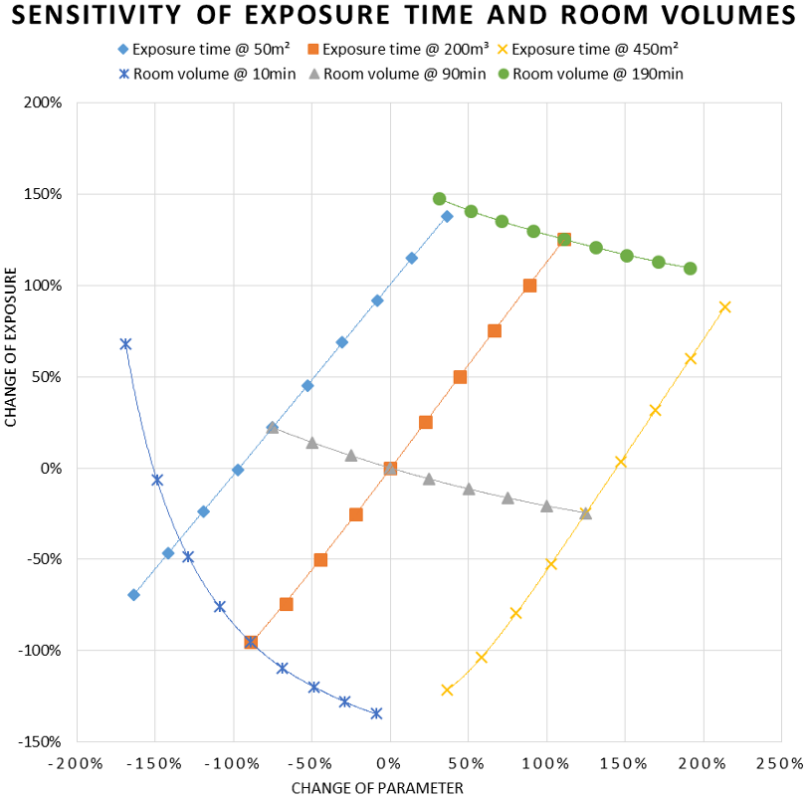
Sensitivity of exposure time and room volume

**Figure 10:**
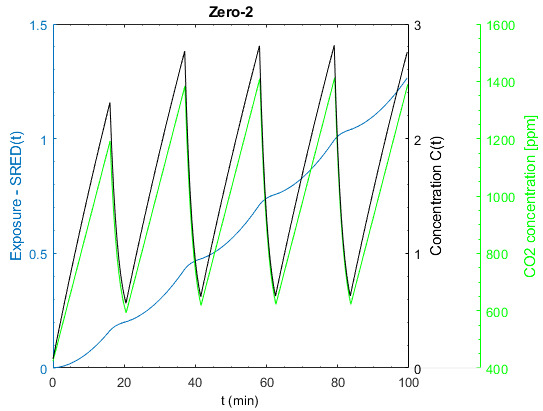
Change of exposure, virus and CO_2_ concentration over time

For the sensitivity analysis of window ventilation, a slightly different zero situation “Zero 2” was defined. Table 6 (Tab. 6) shows the parameter set for situation Zero 2, and Figure 9 (Fig. 9) depicts the modelled results. Figure 10 (Fig. 10) demonstrates the results of the sensitivity analysis, and Table 7 (Tab. 7) contains the absolute numbers of the parameter variations. The window height has an effect on exposure, as the window functions as air inlet and outlet at the same time. Whenever the outdoor temperature is lower than the indoor temperature, the air will flow in through the lower part of the window and out through the upper part. Between these areas is a neutral line with no air flow. The differential pressure driving the air flow increases with increasing distance from the neutral line. Therefore, higher windows or doors have higher CADR than narrower windows with the same total surface. However, this effect seems negligible, as long as the window surface is large enough. The increase of the window surface increases the CADR strongly and causes a log reduction of the exposure. This points out how important the facility conditions are when using window ventilation. 

Only the extension of the total opening duration has a similar effect on the exposure. It reduces the exposure with increasing duration almost in parallel to the increasing window surface. For durations longer than in the Zero 2 situation, the curve slowly starts to show an even stronger decrease of exposure. In any case, we have to keep in mind that, as already explained in the model description, the CADR may be slightly overestimated for long opening periods and large temperature differences. 

The other conspicuous curve is the one for the intervals. The number describes how many different sections into which the total opening time is divided. These intervals are always centered on the time line. The same closing times are used before the first, after the last and in-between all openings. This is the only discrete parameter in this analysis. During the closing intervals, the aerosol concentration is only reduced by the decay of the viability and the very low leakage air flow, which leads to a strong build-up of the concentration in the presence of an infected person. Since the exposure is an integral function of the concentration, the unventilated periods should not be too long, in order to avoid high concentration levels, which contribute strongly to the exposure.

Therefore, the exposure decreases with increasing number of intervals. The decrease is very strong when the amount is increased from one opening to three and becomes a little weaker for further increases. This is because with an increasing number of intervals, the opening time per interval decreases and the reduction of the concentration will stop at relatively high levels. From the thermal point of view, short opening is also preferable on cold days. 

**Figure 11:**
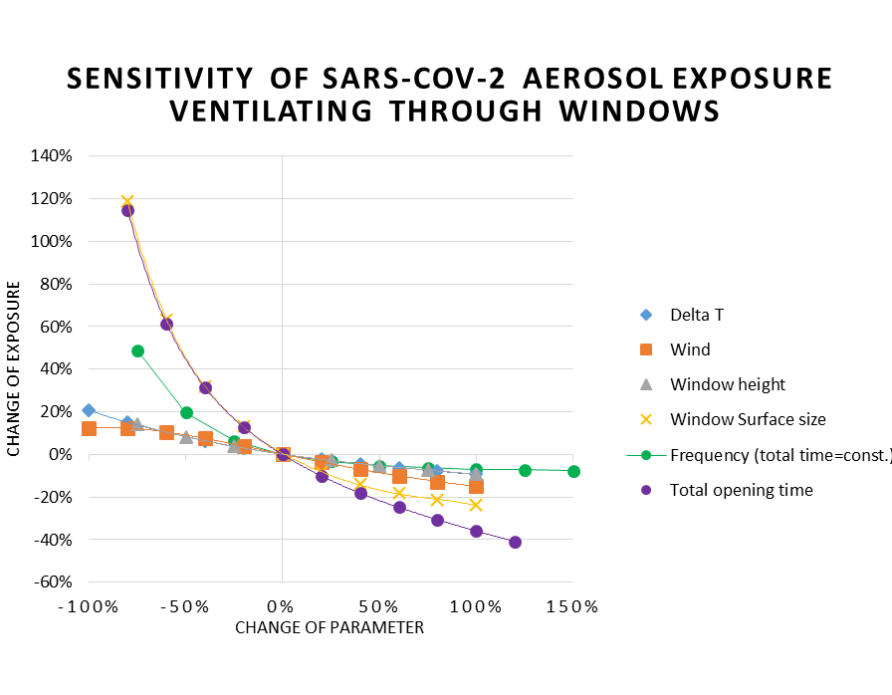
Sensitivity analysis for window ventilation

**Figure 12:**
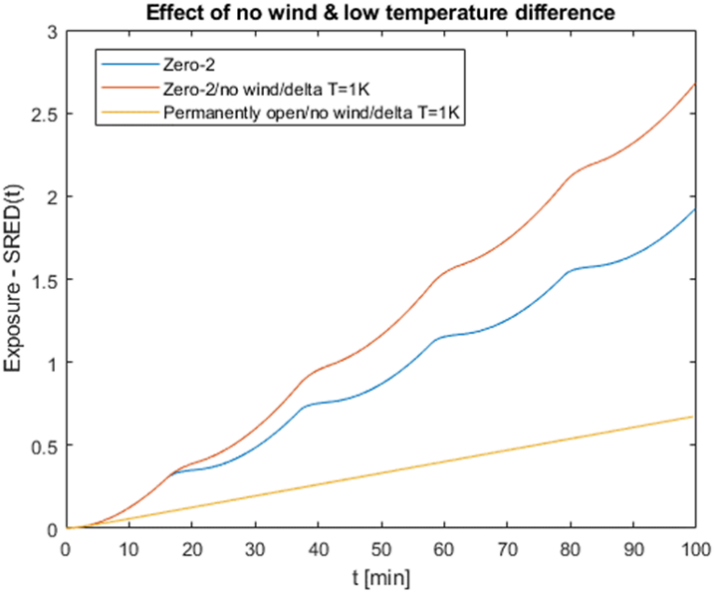
Influence of no wind and low temperature difference for interval and permanent opening of windows

Both wind and temperature differences are the physical forces which force the air to flow through the window. Figure 11 (Fig. 11) shows that the exposure is not very sensitive if one of these strongly declines. The probability that both parameters will fall to zero seems extremely low and if it ever happens, it will probably not last very long. If the outdoor temperature is close to the indoor temperature, interval opening is no longer required for thermal reasons and leaving the window open will increase the CADR significantly. In order to verify that a potential low wind/low temperature difference situation is not as critical as it may seem, we conducted two additional variations of the Zero 2 situation with no wind and low (1 K) temperature difference – one with interval and one with permanent opening – and compared it to the original situation. As Figure 12 (Fig. 12) shows, the exposure increases significantly when there is no wind, the outdoor temperature is similar to the indoor temperature, and the same ventilation strategy is chosen. However, when leaving the windows open, exposure decreases dramatically. This means that leaving the windows open is indicated as soon as the outdoor temperatures approach the indoor temperature or higher. 

## 4. Conclusions

Summarizing the results of the basic parameter sensitivity analysis, it can be said that:

- **Small rooms** significantly increase the exposure. Good ventilation reduces the effect. For very short time periods, the use of (preferably ventilated) small rooms seems acceptable.
- **Poor ventilation** increases the exposure strongly, especially in small rooms. This is only acceptable for very short time periods.
- **Exposure time** has a very strong effect on the exposure. Doubling the exposure time will most likely more than double the exposure. Especially in poorly ventilated rooms, this happens at a high level of exposure. Reducing the time of event seems to be one of the strongest measures to reduce aerosol exposure.
- **Masks** are also a very strong mitigation measure. The effect is the strongest for the first ~50% of mask effectivity. Therefore the use of any kind of masks is highly recommended for indoor settings. Whether or not the obligation to wear professional high quality masks are worth the additional effort must be critically examined
- **The reduction of control measures** will obviously significantly increase the aerosol exposure dose in presence of an infected person

Summarizing the results of the window ventilation parameter sensitivity analysis, it can be said that:

- A sufficient **total window surface** and **total opening time** will increase the fresh air supply and greatly reduce exposure
- The **number of intervals** into which the total opening time is divided should not be too low. Long closing periods need to be avoided.
- **Temperature difference** and **wind** are the driving forces of the fresh air supply. However, exposure is not extremely sensitive to them. In case that both are very small, and keeping the windows open may solve the problem sufficiently in many settings.
- The **window height** affects the exposure, but the sensitivity is not high.

**Figure 13:**
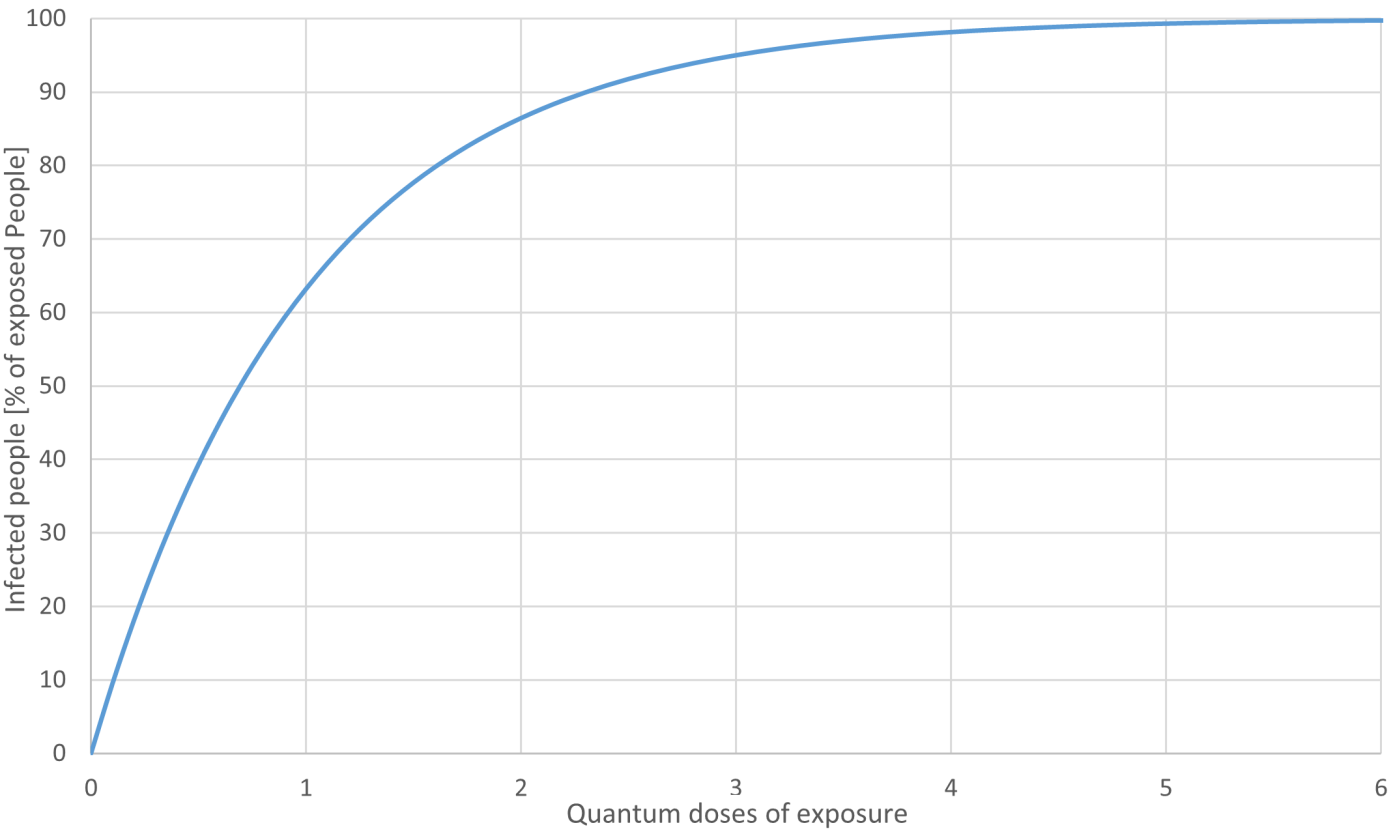
Quantum dose curve

In order to assess the value of exposure reduction measures for specific situations for infection prevention and fatality reduction, further investigations are required. Looking at a quantum dose curve (Figure 13 (Fig. 13)) the question may come up, whether the value of the mitigation measures could reduced due to more infectious variants like delta. The highest value for an exposure reduction can be found at the lower, steep part of the curve, but exposure reduction is lower at the upper end. For instance, a reduction of 50% starting from 0.5 quanta will reduce the number of infections significantly, and a reduction of 50% starting at 4 quanta will prevent fewer infections.

## Notes

### Competing interests

Christian Redder works as a development engineer in the field of aerosol separation at Delbag GmbH (Herne). The manuscript was written independently of this activity, without any company involvement.

## Figures and Tables

**Table 1:**
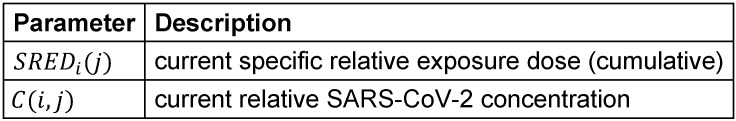
Output values

**Table 2:**
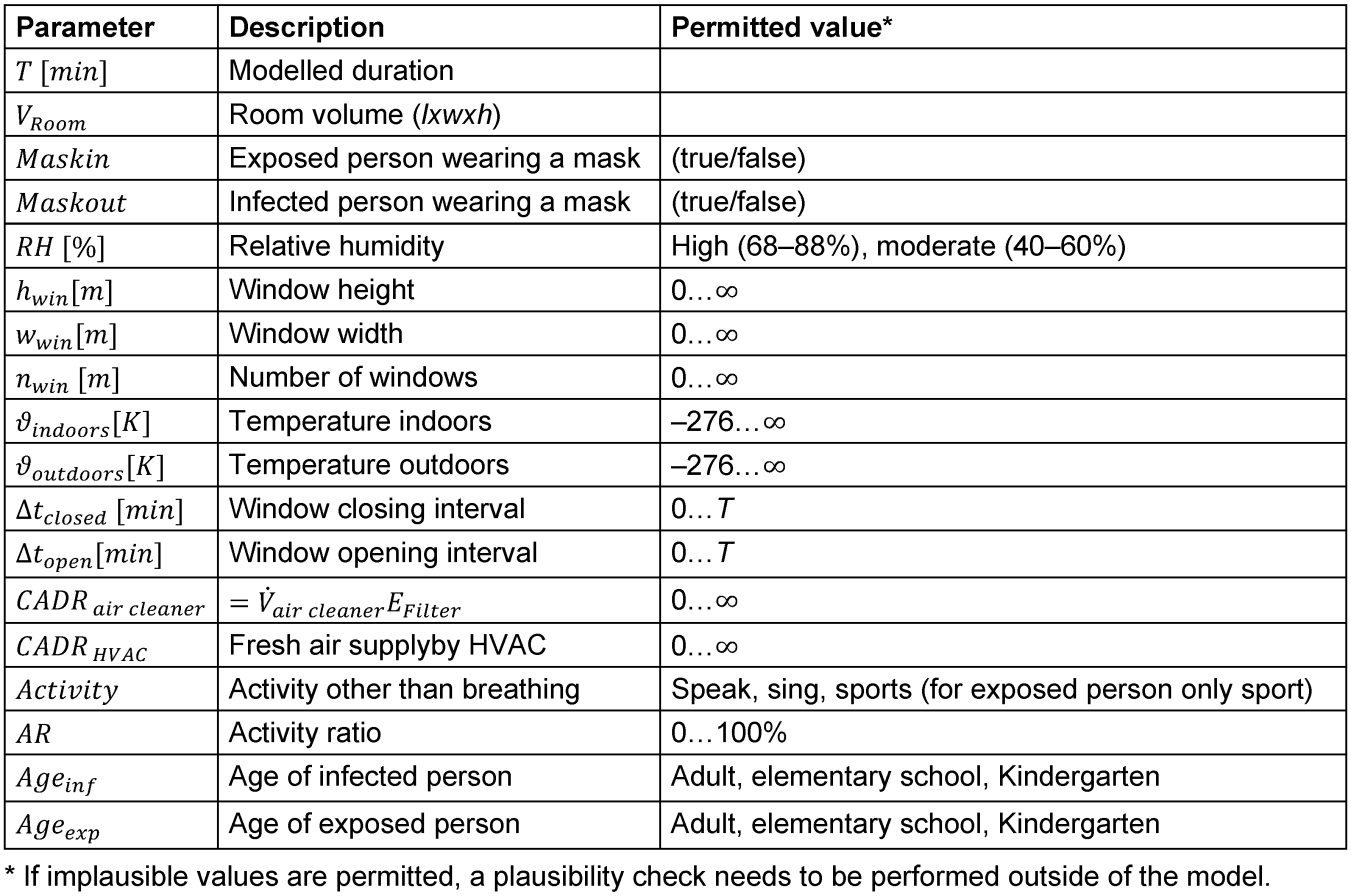
Input parameters

**Table 3:**
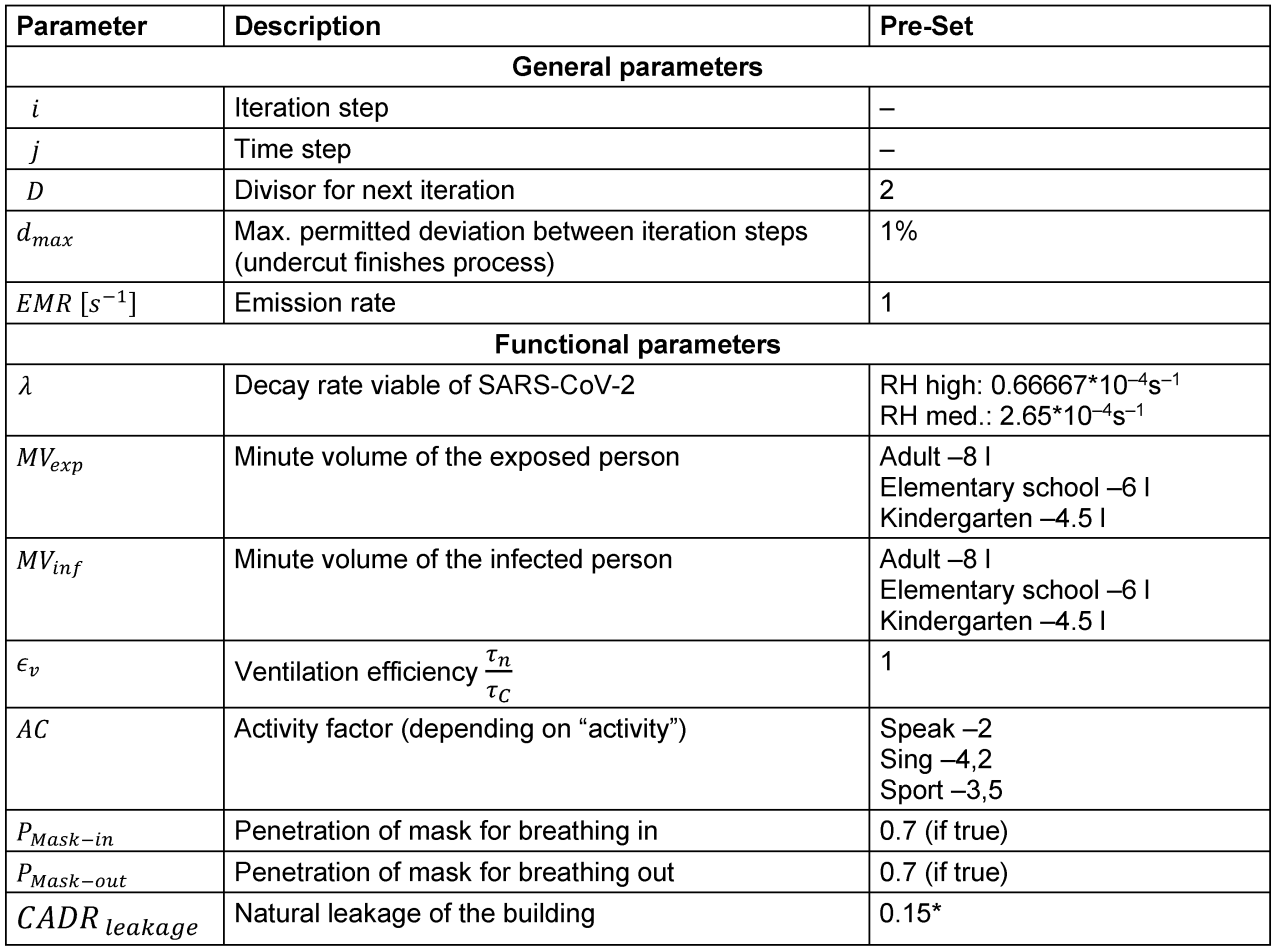
Functional parameter (pre-set)

**Table 4:**
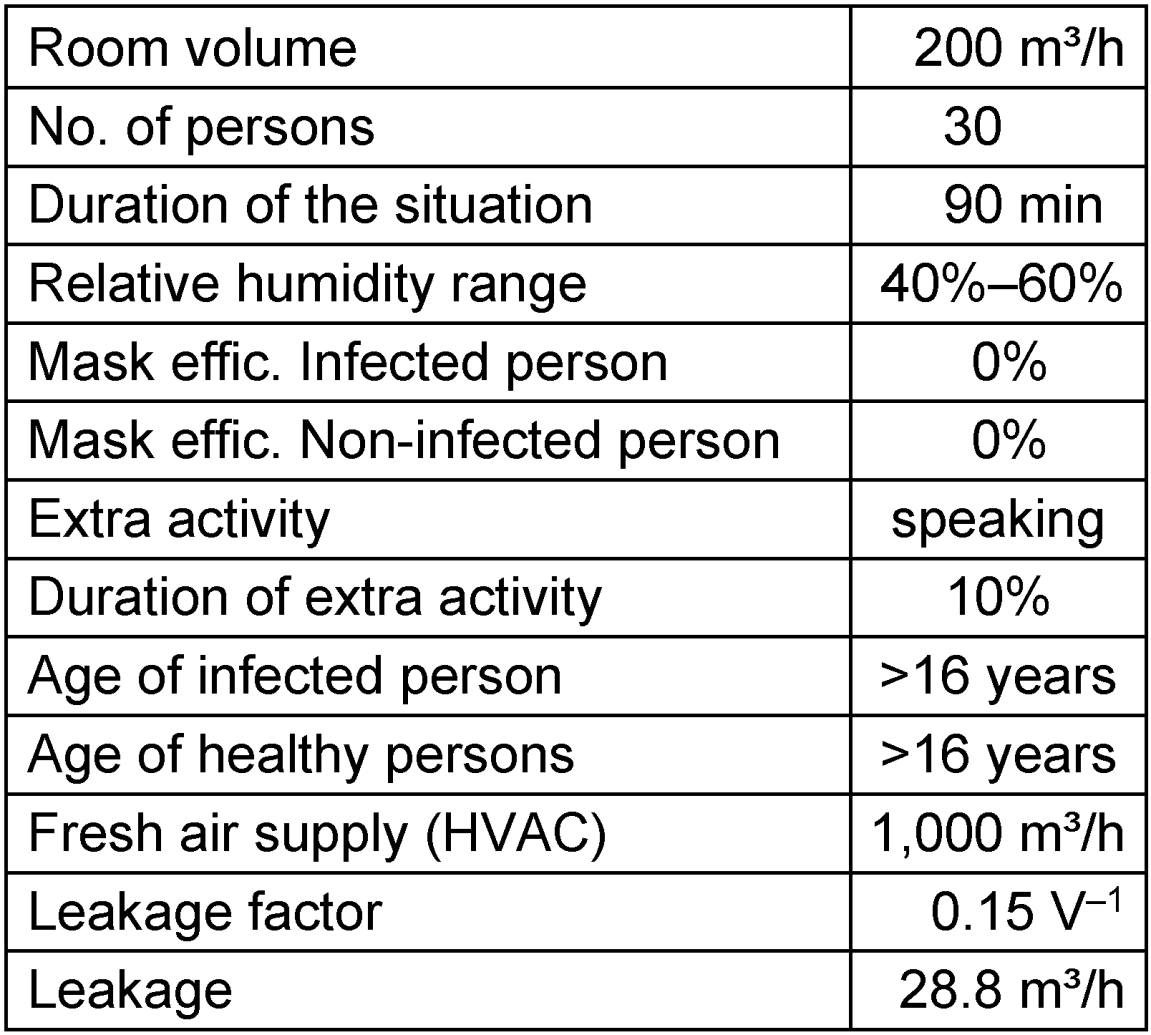
Initial parameters for the sensitivity analysis

**Table 5:**
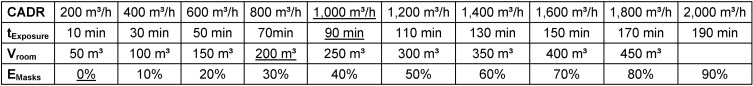
Modelled values in absolute numbers (zero situation is underlined)

**Table 6:**
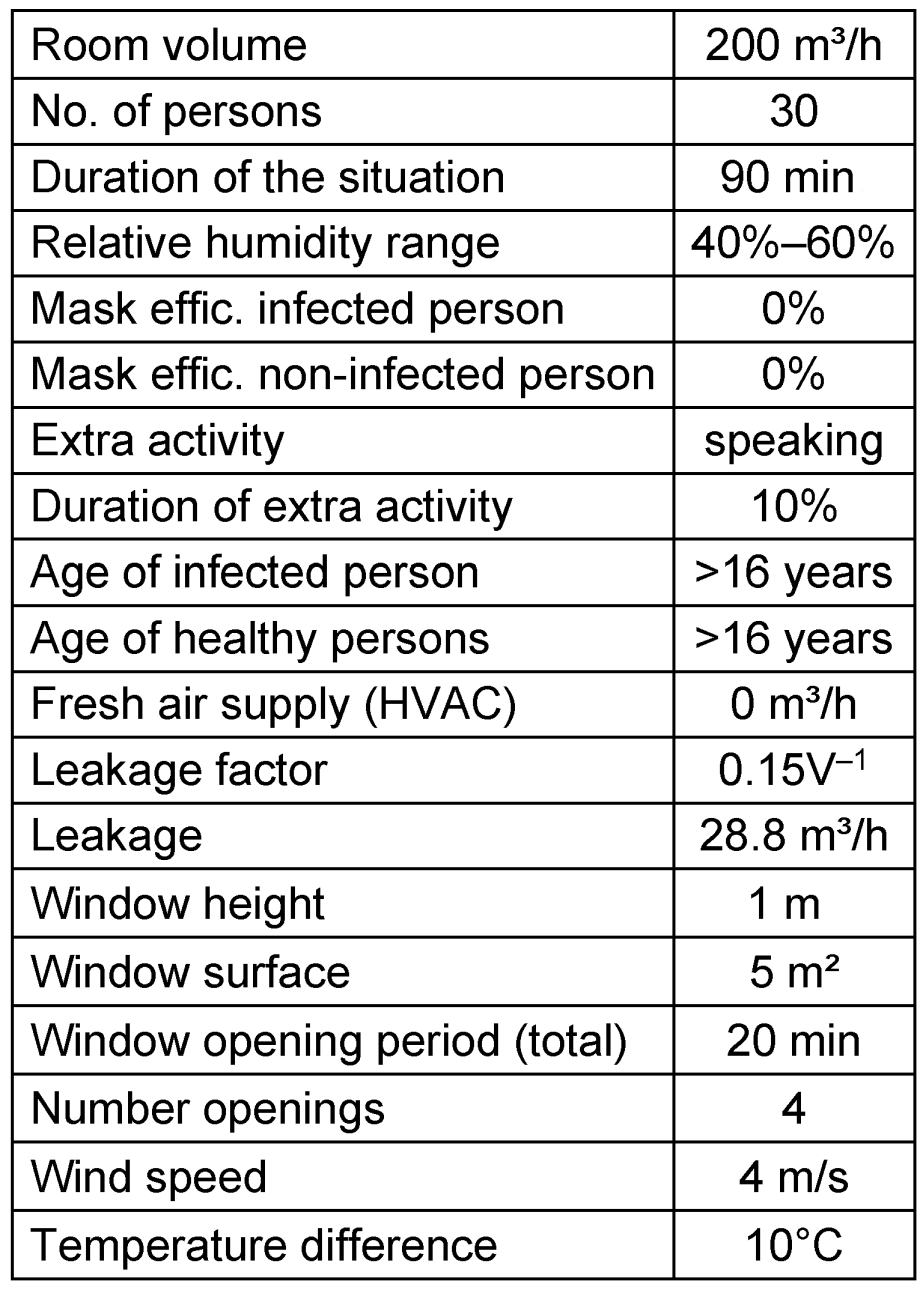
Initial parameters „window ventilation“

**Table 7:**
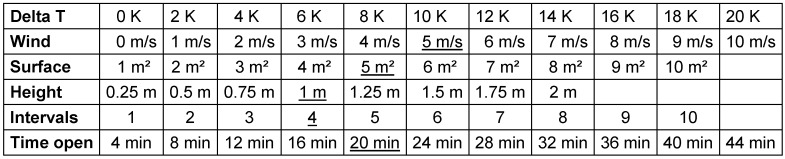
Modelled values in absolute numbers (zero situation is underlined)
